# *Chlamydia*-Like Organisms (CLOs) in Finnish *Ixodes* *ricinus* Ticks and Human Skin

**DOI:** 10.3390/microorganisms4030028

**Published:** 2016-08-18

**Authors:** Kati Hokynar, Jani J. Sormunen, Eero J. Vesterinen, Esa K. Partio, Thomas Lilley, Veera Timonen, Jaana Panelius, Annamari Ranki, Mirja Puolakkainen

**Affiliations:** 1 Department of Virology, Hartman institute, University of Helsinki, Helsinki 00014, Finland; veera.timonen@helsinki.fi (V.T.); mirja.puolakkainen@helsinki.fi (M.P.); 2 Department of Biology, University of Turku, Turku 20014, Finland; jjtsor@utu.fi (J.J.S.); ejvest@utu.fi (E.J.V.); 3 Department of Agricultural Sciences, University of Helsinki, Helsinki 00014, Finland; 4 Medical Center Söder, Söderkulla 01150, Finland; partio@kolumbus.fi; 5 Biology Department, Bucknell University, Lewisburg, PA 17837, USA; tmlill@utu.fi; 6 Department of Dermatology and Allergology, University of Helsinki and Helsinki University Central Hospital, Helsinki 00250, Finland; jaana.panelius@hus.fi (J.P.); annamari.ranki@hus.fi (A.R.)

**Keywords:** *Chlamydiales*, *Chlamydia*-like organisms (CLOs), ticks, phylogeny, 16S rRNA, PCR, skin

## Abstract

Ticks carry several human pathogenic microbes including *Borreliae* and *Flavivirus* causing tick-born encephalitis. Ticks can also carry DNA of *Chlamydia*-like organisms (CLOs). The purpose of this study was to investigate the occurrence of CLOs in ticks and skin biopsies taken from individuals with suspected tick bite. DNA from CLOs was detected by pan-*Chlamydiales*-PCR in 40% of adult ticks from southwestern Finland. The estimated minimal infection rate for nymphs and larvae (studied in pools) was 6% and 2%, respectively. For the first time, we show CLO DNA also in human skin as 68% of all skin biopsies studied contained CLO DNA as determined through pan-*Chlamydiales*-PCR. Sequence analyses based on the 16S rRNA gene fragment indicated that the sequences detected in ticks were heterogeneous, representing various CLO families; whereas the majority of the sequences from human skin remained “unclassified *Chlamydiales*” and might represent a new family-level lineage. CLO sequences detected in four skin biopsies were most closely related to “uncultured *Chlamydial* bacterium clones from *Ixodes* *ricinus* ticks” and two of them were very similar to CLO sequences from Finnish ticks. These results suggest that CLO DNA is present in human skin; ticks carry CLOs and could potentially transmit CLOs to humans.

## 1. Introduction

A total of nine families have so far been recognised as members of the order *Chlamydiales* [1,2]. The most widely studied is the *Chlamydiaceae*-family that includes the well-known human pathogens *Chlamydia trachomatis* and *C. pneumoniae*, as well as several animal pathogens (some with zoonotic potential). Members of the remaining eight families (*Clavichlamydiaceae*, *Criblamydiaceae*, *Piscichlamydiaceae*, *Parachlamydiaceae*, *Rhabdochlamydiaceae*, *Simkaniaceae*, *Waddliaceae*, and *Parilichlamydiaceae*) are called *Chlamydia*-like organisms (CLOs). They share intracellular lifestyle, biphasic developmental cycle and a large core gene set (the “Pan-Genome of the *Chlamydiae*”) with the genus *Chlamydia* [3]. A variety of CLOs have been detected in various environmental (water and soil) samples, in amoebae and in animals, such as bats, deer, seabirds, snakes, arthropods, isopods and fish [2,4,5,6,7,8,9,10]. The role of CLOs as human pathogens is currently being explored: Recent publications have reported association between *Waddlia chondrophila* and tubal factor infertility [11], adverse pregnancy outcome [12] and lower respiratory tract infections [13]; *Simkania negevensis* and *Rhabdochlamydia* spp. may be associated with respiratory infections [14,15,16], and *Parachlamydia acanthamoebae* with pneumonia [17,18,19].

*Ixodes ricinus*, the most common species of tick in Europe, is known to carry and transmit several microbes pathogenic to animals and humans, including *Borrelia burgdorferi* sensu lato, the causative agent of Lyme disease, *B. miyamotoi*, causing relapsing fever, *Anaplasma phagocytophilum*, the etiologic agent of human anaplasmosis, and *Babesia* sp. causing babesiosis [20,21,22]. In addition, *I. ricinus* and *Ixodes persulcatus* can transmit tick-borne encephalitis (TBE) virus. The life cycle of the *Ixodes* tick involves four stages: egg, larva, nymph and adult. The *Ixodes* tick needs a blood meal during every post-hatching life stage, and thus needs to find a new host at each stage. Therefore, *I. ricinus* might transmit pathogens forward during the subsequent meal. While some *Ixodes* species are host specific, *I. ricinus* feeds on various species including humans. In Europe, *I. ricinus* is the most common vector known to transmit a variety of pathogenic microbes to humans, and ticks have also been shown to be carriers of CLO DNA. In Switzerland, a substantial number of ticks were collected and studied for *Chlamydial* DNA by a PCR method amplifying a fragment of the 16S rRNA gene [7,23]. In both studies, ticks were found to carry DNA of members from several families of the *Chlamydiales* order. Two-thirds of the sequenced samples belonged to the *Rhabdochlamydiaceae* and the *Parachlamydiaceae* families [23].

The aim of this study was to investigate the prevalence of CLOs in ticks and in skin. Consequently, we analysed more than 1800 *Ixodes* ticks (in pools and individually) collected from southwestern Finland for the presence of CLOs by PCR. To seek evidence of possible transmission of CLOs to human via tick bite, skin biopsies screened for *Borrelia burgdorferi-*specific DNA obtained from individuals with suspected tick-related skin manifestation and skin biopsies from healthy individuals were analysed.

## 2. Materials and Methods

### 2.1. Ticks and DNA Extraction

Questing *Ixodes ricinus* (Acari: Ixodidae) of all life stages were collected in an earlier study by blanket dragging from May to September in 2012 and 2013 [20,24]. Ticks (*n* = 1823) were collected from two rural islands, Seili and Boskär, located in the inner archipelago of the Archipelago Sea in southwestern Finland. Southwestern Finland is a region endemic to Lyme borreliosis spirochetes, where increasing tick abundance has recently been observed [20,24,25]. Furthermore, three adult *Ixodes persulcatus* samples were provided by Ritva Penttinen (Zoological Museum, University of Turku, Turku, Finland) for analysis. Total DNA was extracted from collected tick samples (*n* = 1826) using NucleoSpin^®^ TriPrep-kits (Macherey-Nagel, Düren, Germany), following the protocol in the kit manual. More comprehensive description of tick sampling, DNA extraction, and other pathogens found in these ticks can be found in Sormunen, et al. [20,24].

### 2.2. Skin Samples and DNA Extraction

To gain support on whether the CLOs could be transmitted from ticks to humans through a tick bite, we studied a total of 80 archived DNA samples extracted from diagnostic skin biopsies obtained from patients with a suspected tick-borne skin reaction [26]. All above skin biopsies were obtained for routine histopathological analysis and *Borrelia burgdorferi* sensu lato DNA detection by PCR methods (16S rRNA and *ospA* as targets) as described earlier [27,28]. Of the specimens from individuals with suspected tick bite, 39 with PCR-confirmed *B. burgdorferi* infection and 41 PCR-negative were included in this study. As a control group, we studied 39 archived DNA samples extracted from healthy skin [29,30]. These samples were obtained from healthy adults during arthroscopy due to joint trauma (a small sample of skin was simultaneously collected from the arthroscopy wound edge at Dextra Medical Center, Helsinki, Finland), and from healthy hospital or laboratory staff (a punch biopsy). All subjects gave their informed consent for inclusion before they participated in the study. The study was conducted in accordance with the Declaration of Helsinki, and the protocol was approved by the Ethics Committee of Helsinki and Uusimaa Hospital District (Dnro HUS 553/E6/01).

### 2.3. DNA Amplification and Sequencing

A total of 326 DNA samples extracted from ticks and pooled ticks, and 119 samples extracted from skin were screened for the presence of CLOs, by using a *Chlamydiales*-specific Pan-*Chlamydiales* real-time TaqMan PCR targeting the 16S rRNA-encoding gene [31]. This PCR method amplifies an approximately 200 bp fragment of the 16S rRNA-encoding gene, and has been shown to detect a wide range (at least all the 15 *Chlamydial* reference strains tested) of different members of the *Chlamydiales* order, and sensitive for at least five DNA copies per reaction of the positive control (with an efficiency of 75%). A PCR reaction of 25 μL contained 12.5 μL Maxima Probe/ROX qPCR Master Mix (2X) (Thermo Scientific, Waltham, MA, USA), 100 nM of primers (panCh-Fwd: 5′-ccgcaacactgggact-3′, panCh-R: 5′-ggagttagccggtgcttctttac-3′) and 100 nM probe (panCh-Probe: 5′-FAM-ctacgggaggctgcagtcgagaatc-BHQ1). Primers and probe were purchased from Integrated DNA technologies. Amplification was performed with 7500 Real-Time PCR system (Applied Biosystems). Cycling conditions were 50 °C/2 min, 95 °C/10 min, and 45 cycles of 95 °C/15 s, 60 °C/1 min.

Sequence analysis of 16S rRNA-encoding gene is a widely used preliminary method for bacterial species classification and identification. Thus, we also amplified and sequenced regions of *Chlamydiales* 16S rRNA-encoding gene from ticks and skin biopsies, and compared those to the reference sequences deposited in GenBank. Amplicons of the Pan-*Chlamydiales* qPCR were purified by Illustra ExoProStar 1-Step (GE Healthcare, Buckinghamshire, UK) as instructed by the manufacturer and sequenced using primer panFseq [31]. Sequencing was performed in the sequencing unit of Institute for Molecular Medicine Finland (https://www.fimm.fi/en/services/technology-centre/sequencing). BLAST analysis was performed in order to compare the gained CLO sequences to the known sequences in NCBI database. Sequence data has been deposited into the NCBI database (GenBank) and the accession numbers are provided in Table S1. A maximum likelihood tree was constructed of the 16S rRNA sequences and is shown in Figure S1 [32,33].

## 3. Results

### 3.1. Prevalence and Sequence Analysis of Chlamydia-Like Organisms (CLOs) in Ticks Collected from Finland

Altogether 326 DNA samples extracted from individual ticks and pooled ticks were analysed by Pan-*Chlamydiales* PCR targeting the 16S rRNA gene. CLO prevalence was 40% for adult ticks (19/47) (Table 1).

The minimum infection rate (number of positive pools/total number of ticks) was 6.0% (30/497) for pooled nymph samples (215 pools, 1–14 individuals per pool, altogether 497 nymphs) and 1.7% (22/1282) for pooled larval samples (163 pools, 1–139 individuals per pool, altogether 1282 larval ticks).

**Figure 1:**
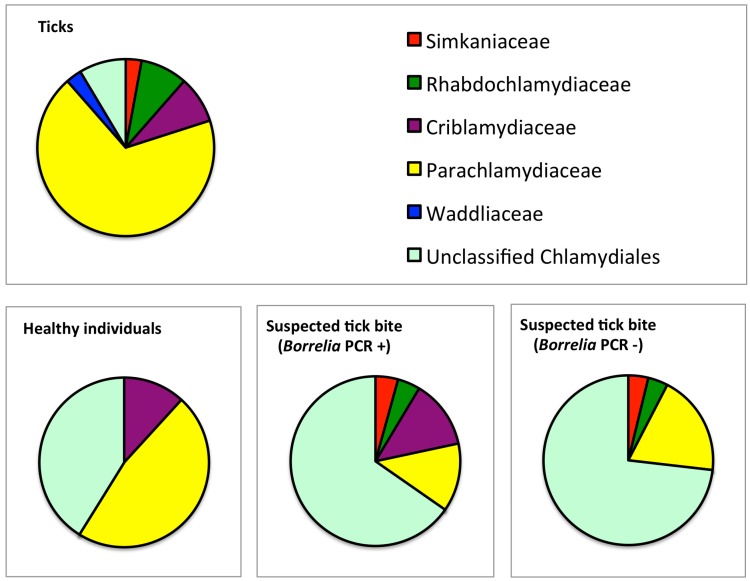
*Chlamydiales* families in the sequenced pan-*Chlamydiales* PCR-positive tick and skin samples. CLOs belonging to the *Parachlamydiaceae* family were the most common family-level lineage in ticks. Unclassified *Chlamydiales* were prevalent in skin biopsies.

All PCR products were sent for sequencing, and readable sequence (approximately 170 bp) data was obtained from 35 samples. Classification criteria (cut-offs of 97%, 95% and 90% of sequence identity within the 16S rRNA gene can be used for designation of species, genus and family levels of the *Chlamydiales* order, respectively) published by Everett [1] were used to classify CLOs [7,23]. When compared to *Chlamydiales* 16S rRNA gene sequences, 32 tick-derived CLO sequences showed above 90% identity with the earlier described strains allowing identification at the family level (Figure 1, Table 2).

The majority of the sequences belonged to the *Parachlamydiaceae* family (24 sequences, 69%) (Figure 1). Three (9%) sequences belonged to *Rhabdochlamydiaceae* and 3 (9%) to *Criblamydiaceae* family (Table 2). Interestingly, the best BLAST match for only two sequences (6%) was JQ860079, “uncultured *Chlamydiales* bacterium isolated from tick *Ixodes ricinus.*” The best BLAST match for a majority of the sequences was “uncultured *Chlamydiales* bacterium clone” from water sources, such as domestic shower heads, raw surface water, and amoebae (14/35, 40%), and “nasopharyngeal samples from hospitalized children” (12/35, 34%). Identity percentages and the GenBank accession numbers of the best BLAST hits are shown in Table S1.

### 3.2. Prevalence and Sequence Analysis of CLOs in Human Skin

**Figure 2:**
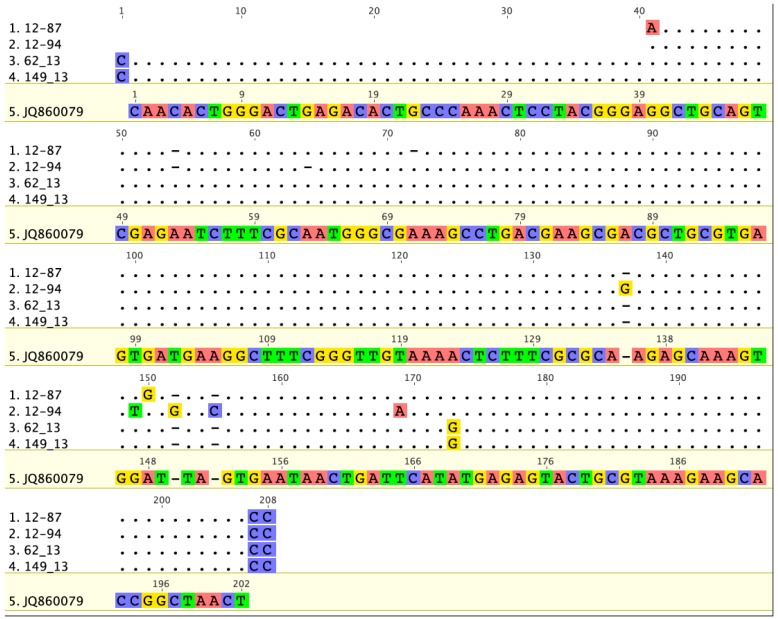
Comparative analysis of the CLO 16S rRNA sequences from ticks (63_13 and 149_13) and two skin biopsies (12–87 and 12–94). The sequences are 95%–98% similar. The best BLAST match for tick CLO sequences (JQ86007, from *Ixodes ricinus*) is shown as a reference. *Geneious version 6.1 created by Biomatters. Available from http://www.geneious.com*.

CLO DNA was detected in human skin, and the prevalence was higher in specimens from individuals with suspected tick bite (62/80, 78%) than in healthy skin (19/39, 49%). The pan-*Chlamydiales* PCR-positive samples (*n* = 81) were sent for sequencing, and sequences were obtained from 66 samples (Table S1). BLAST analysis revealed that four skin samples contained sequences that matched best with the GenBank sequences from “uncultured *Chlamydiales* bacterium clone” from *Ixodes ricinus*. Two of these sequences belonged to family *Parachlamydiaceae* and two to *Rhabdoclamydiaceae*. In addition, two of the tick-derived CLO sequences (62_13 and 149_13, Table S1) belonging to the family *Rhabdochlamydiaceae* were 95%–98% similar to the *Rhabdochlamydiaceae* CLO sequences from skin (12–87 and 12–94, Figure 2, Table S1).

Sequences belonging to *Rhabdochlamydiaceae* or *Simkaniaceae* families were not detected in biopsies from healthy individuals (Figure 1, Table 3). Sequencing results showed that a majority (62%) of CLO sequences in skin showed < 90% identity not allowing classification at the family level. Again, “uncultured *Chlamydiales* bacterium clones” mainly from various water sources, were the best BLAST matches for the CLOs from skin. The best BLAST matches with accession numbers and identity percentages are presented in detail in Table S1.

## 4. Discussion

The first recovery of *Chlamydial* organisms (called psittacosis-lymphogranuloma venereum agents at that time) in ticks was reported in 1969 [34]. Our study investigated the presence and diversity of *Chlamydia*-like organisms (CLO) DNA in ticks and in human skin with pan-*Chlamydiales*-PCR and sequence analysis of the PCR product. Altogether, 40% of the investigated adult ticks, at minimum 3% of the tick pools, and 68% of all the human skin biopsies studied were positive for CLO DNA. To our knowledge, this is the first time CLOs have been examined and found in human skin biopsies. The estimated prevalence of *Chlamydiales* DNA in *Ixodes ricinus* in an earlier study was 4%–28% [7], but a later study consisting of a larger collection of ticks estimated only < 1% of individual ticks to carry *Chlamydial* genetic material [23]. Of ticks harvested in Algeria, as much as 45% were shown to contain *Chlamydial* DNA [7]. In addition to rather high prevalence, a diversity of CLOs was observed in ticks. Among the ticks collected from southwestern Finland, most CLO sequences belonged to *Parachlamydiaceae* (74%), whereas a smaller proportion contained *Rhabdochlamydaceae* DNA (9%). Similarly, in a large number of ticks collected in Switzerland and in Algeria, the most prevalent CLO sequences detected belonged to the family *Parachlamydiaceae* (33%) and *Rhabdoclamydiaceae* (29%). The observed differences in prevalence and diversity may be due to differences in investigation strategy and methods: In the Swiss studies, ticks were analysed in large pools, and the prevalence is an estimate, whereas a portion of the Finnish and the Algerian ticks were examined individually, albeit in smaller number. Moreover, differences in the environment, including host animals of the ticks, could partly explain this.

Transmission of *Chlamydia* to human via tick bite was indirectly suggested earlier by development of antibodies against *Chlamydia* after tick bite in children [35]. To assess whether CLOs could be transmitted to humans via ticks, we studied DNA extracted from skin biopsies taken from individuals with suspected tick bite in history. CLOs could be detected in 85% of the skin biopsies from *B. burgdorferi* PCR-positive and 71% from *B. burgdorferi* PCR-negative lesions. Moreover, the two *Rhabdochlamydiaceae* sequences from skin specimens showed 95%–98% similarity with the sequences from Finnish ticks. This suggests that ticks, indeed, could serve as a vector of transmission. Very little is known about the clinical relevance of CLOs in general and thus, practically nothing can be said about the significance of these findings. Also, specimens from healthy individuals contained CLO DNA. This is not surprising as recent microbiome studies have shown that skin carries DNA from various microbes [36,37], although *Chlamydiales* DNA as such was not detected in these studies. As a matter of fact, tick bites often go unnoticed, so we cannot exclude tick exposure in the healthy individuals. Indeed, *Borrelia miyamotoi* infection does not necessarily cause erythema migrans-like skin symptoms [38]. However, most of the sequences observed in healthy skin were related to water-associated CLO sequences. Indeed, CLOs are found in the environment and various water sources [39,40,41,42]. The sequence analysis of CLO DNA was performed of a highly variable ≤ 200 bp fragment of the 16S rRNA gene [7,31]. Criteria proposed by Everett [1] were used to putatively classify the sequences at the family-level and genus-level lineages [7]. A majority (62%) of CLO sequences in skin showed < 90% identity with established *Chlamydial* strains and were unclassified *Chlamydiales*. Although the discriminatory power of this approach can be limited, we suggest that CLO sequences in skin represent largely unknown, potentially novel family-level lineage(s) in *Chlamydiales*. As more whole genome sequences of CLOs are determined, more precise analyses can be performed. We cannot yet answer whether CLO DNA is associated with pathogenesis of skin disorders or whether CLO DNA stays in the skin after acquisition, like DNA from ssDNA viruses remains as a bioportfolio [29].

It is not known how and where CLOs are acquired and how they end up in human skin. The occurrence of CLOs in wild mammals in Finland, potential hosts for ticks, has thus far been poorly investigated. Earlier studies have shown that bats can be a reservoir for a variety of pathogens, including *Bartonella* species [43,44] and viruses [45,46]. Our earlier study shows that CLO DNA is found in bats (*Myotis daubentonii*) and their prey insects [10]. Phylogenetic analysis suggested that 56% of the CLO sequences obtained from bats and 39% of those from insects belong to the family *Rhabdochlamydiaceae* [10]. However, most tick-derived sequences did not assemble together with the bat-associated CLO sequences, suggesting that the majority of CLOs in ticks observed in this study did not originate from bats (data not shown). Major hosts of ticks in the wild include small rodents and deer [47,48], which should be the next avenue of research to pursue. We also showed that insects carry CLO DNA and sequences belonging to families *Rhabdochlamydiaceae* and *Parachlamydiaceae* were most common [10]. Thus, other arthropods besides ticks could also serve as a vector for CLOs and spread the bacterium to human skin through bites.

Sequences identical to or resembling most closely the *Chlamydia* genus were not detected in ticks. This confirms the earlier notion that *C. trachomatis* is a human pathogen and likely transmitted only between humans with the exception of flies that can carry *C. trachomatis* DNA in trachoma-endemic area [49]. Other species belonging to the genus *Chlamydia* were not identified in ticks either, although some of them are animal pathogens. Neither *C. trachomatis* nor *C. pneumoniae* DNA was detected in the skin biopsies studied here. Contradictory evidence of *Chlamydia*-specific DNA sequences in some conditions, such as mycosis fungoides and keratoderma blenorrhagicum, has been presented [50,51,52].

In conclusion, CLO DNA was frequently detected in human skin and ticks in Finland. Our findings are in agreement with the earlier studies showing that ticks carry CLO DNA. Moreover, our results show that CLOs can be detected in human skin, and a remarkable sequence similarity was observed between sequences from ticks and skin. The transmission routes of CLOs remain unknown, but ticks can represent a transmitting vector. The significance of CLOs in skin remains to be investigated.

## Figures and Tables

**Table 1:** Prevalence of *Chlamydiales* DNA in *Ixodes* tick life stages.

Tick Life Stage	No. of Ticks	No. of Positive Specimens ^1^	No. of Samples Examined ^2^	No. of Positive Samples/Total No. of Individuals (Prevalence of CLO DNA in Individual Ticks %)	No. of Positive Samples/ Total No. of Individuals (Minimum Infection Rate ^3^)
Adult	47	19	47	19/47 (40.4%)	
Nymph	497	30	215		30/497 (6.0%)
Larva	1282	22	63		22/1282 (1.7%)
Total	1826	71	325		52/1779 (2.9%)

^1^ Five adult ticks and ten nymph pools also carried *Borrelia* DNA [24]; ^2^ Adult samples contained a single individual; nymph and larval samples were pools that contained 1–139 individuals; ^3^ Number of positive pools/total number of ticks.

**Table 2:** Sequencing results and classification of the 16S rRNA gene fragment of pan-*Chlamydiales* PCR-positive tick samples (*n* = 35).

Family-Level (≥90%) ^1^	Genus-Level (≥95%) ^1^	Species-Level (≥97%) ^1^
*Parachlamydiaceae* (*n* = 24)	*Parachlamydia* (*n* = 8)	*Parachlamydia acanthamoebae* (*n* = 2)
	*Neochlamydia* (*n* = 4)	*Neochlamydia* sp.
	*Protochlamydia* (*n* = 1)	Trut23-12-2015_Venoge-Embouchure
	*Candidatus Metachlamydia* (*n* = 1)	(*n* = 1)
	ND (*n* = 10)	ND (*n* = 11)
*Rhabdochlamydiaceae* (*n* = 3)	*Rhabdochlamydia* (*n* = 3)	*Candidatus Rhabdochlamydia porcellionis* strain 15C (*n* = 2) ND (*n* = 1)
*Criblamydiaceae* (*n* = 3)	ND (*n* = 3)	
*Waddliaceae* (*n* = 1)	ND (*n* = 1)	
*Simkaniaceae* (*n* = 1)	ND (*n* = 1)	
*Chlamydiaceae*	0	
Unclassified *Chlamydiales* (*n* = 3)		

^1^ Taxonomy cutoffs defined by Everett et al. [1]. and applied to classification of CLOs by Pilloux et al. [23]. ND = taxonomic classification could not be determined.

**Table 3:** *Chlamydiales* family-level lineages based on sequencing of the 16S rRNA gene fragment of pan-*Chlamydiales* PCR-positive skin biopsies (*n* = 66). Genus- and species-level information is shown in footnotes (if the level of classification could be determined).

Family-Level Lineage ^1^	Skin Condition (*n*)		
	Suspected Tick Bite (*Borrelia* PCR +) (*n* = 23)	Suspected Tick Bite (*Borrelia* PCR −) (*n* = 26)	Healthy Skin (*n* = 17)
*Parachlamydiaceae*	3	4	8 ^5^
*Criblamydiaceae*	3		2 ^6^
*Rhabdochlamydiaceae*	1 ^3^	2 ^4^	
*Simkaniaceae*	1	1	
*Chlamydiaceae*	0	0	0
Unclassified *Chlamydiales* ^2^	15	19	7

^1^ Taxonomy cutoffs defined by Everett et al. [1] and applied to classification of CLOs by Pilloux et al. [23]; ^2^ CLO sequences showed <90% identity not allowing classification at the family level [1]; ^3^ Genus: *Rhabdochlamydia* (*n* = 1); ^4^ Genus: *Rhabdochlamydia* (*n* = 1), *Candidatus* Rhabdochlamydia porcellionis strain 15C (*n* = 1); ^5^ Genus: *Parachlamydia* (*n* = 2); ^6^ Genus: *Estrella* (*n* = 1).

## Supplementary Materials

The following are available online at www.mdpi.com/2076-2607/4/3/28/s1, Figure S1: Phylogenetic tree of the 16S rRNA sequences analyzed in this study, Table S1: Sequencing results of positive pan-*Chlamydiales* (16S rRNA) qPCR tick and skin specimens collected in Finland.
