# Correction: Hokynar, K. et al. *Chlamydia*-Like Organisms (CLOs) in Finnish *Ixodes ricinus* Ticks and Human Skin. *Microorganisms* 2016, *4*, 28

**DOI:** 10.3390/microorganisms7020060

**Published:** 2019-02-23

**Authors:** Kati Hokynar, Jani J. Sormunen, Eero J. Vesterinen, Esa K. Partio, Thomas Lilley, Veera Timonen, Jaana Panelius, Annamari Ranki, Mirja Puolakkainen

**Affiliations:** 1 Department of Virology, Hartman Institute, University of Helsinki, 00014 Helsinki, Finland; mirja.puolakkainen@helsinki.fi; 2 Department of Biology, University of Turku, 20014 Turku, Finland; jjtsor@utu.fi (J.J.S.); ejvest@utu.fi (E.J.V.); 3 Department of Agricultural Sciences, University of Helsinki, 00014 Helsinki, Finland; 4 Medical Center Söder, 01150 Söderkulla, Finland; ejvest@utu.fi; 5 Biology Department, Bucknell University, Lewisburg, PA 17837, USA; tmlill@utu.fi; 6 Department of Dermatology and Allergology, University of Helsinki and Helsinki University Central Hospital, 00250 Helsinki, Finland; jaana.panelius@hus.fi (J.P.); annamari.ranki@hus.fi (A.R.)

The authors wish to make the following modification to this paper [1]. The forward primer sequence in page 3 should read: (panCh-Fwd [5′-ccgccaacactgggact-3′].

We want to emphasize that this is a typographical error: the sequence of the forward primer used in this study was as indicated in the original publication (Lienard et al. 2011) [1] and in the corrected text.

The manuscript will be updated, and the original will remain online on the article webpage, with a reference to this Correction.
