# Supplementary material for: Chlamydia-Like Organisms (CLOs) in Finnish Ixodes ricinus Ticks and Human Skin
Source: Microorganisms. 2016 Aug 18;4(3):28. doi: 10.3390/microorganisms4030028 (PMC5039588; doi:10.3390/microorganisms4030028)
Supplement: Supplementary file 1 [file microorganisms-04-00028-s001.docx]

**Table S1.** Sequencing results of positive pan-*Chlamydiales* (16S rRNA) qPCR tick and skin specimens collected in Finland.

| **Tick Number** | **GenBank Accession No.** | **Best BLAST Hit** | **Accession** | **Identity %** | **Host** | **Most Identical established *Chlamydiales* Strain** | **Accession** | **Identity %** |
| --- | --- | --- | --- | --- | --- | --- | --- | --- |
| 132_13 | KX451033 | Candidatus Mesochlamydia elodeae strain KV | JN112799 | 92 | free-living amoebae | Candidatus Mesochlamydia elodeae strain KV | JN112799 | 92 |
| 36_13 | KX451010 | Uncultured Chlamydiales bacterium clone GE11061 | HQ721207 | 94 | nasopharyngeal samples from hospitalized children | Candidatus Mesochlamydia elodeae strain KV | JN112799 | 93 |
| 139_13 | KX451035 | Uncultured Chlamydiales bacterium clone GE11061 | HQ721207 | 93 | nasopharyngeal samples from hospitalized children | Candidatus Mesochlamydia elodeae strain KV | JN112799 | 92 |
| 120_13 | KX451030 | Uncultured Chlamydiales bacterium clone 39IR | KF651170 | 92 | bovine rectal swab | Candidatus Mesochlamydia elodeae strain KV | JN112799 | 91 |
| 80_13 | KX451015 | Candidatus Metachlamydia lacustris strain CHSL | GQ221847 | 95 | amoeba | Candidatus Metachlamydia lacustris strain CHSL | GQ221847 | 95 |
| 104_13 | KX451022 | Uncultured Chlamydiae bacterium clone MD2896-0.1m.78 | DQ996922 | 90 | South China Sea sediment | Candidatus Metachlamydia lacustris strain CHSL | GQ221847 | 89 |
| 46_13 | KX451011 | Candidatus Protochlamydia sp. CRIB40 | FJ532293 | 95 | biofilm from clarifier | Candidatus Protochlamydia sp. CRIB40 | FJ532293 | 95 |
| 1_13 | KX451005 | Uncultured Chlamydiales bacterium clone HE210011water | JX083107 | 90 | domestic shower water | Neochlamydia endosymbiont of Acanthamoeba | KF924590 | 89 |
| 109_14 | KX451024 | Uncultured Chlamydiales bacterium clone HE210011water | JX083107 | 91 | domestic shower water | Neochlamydia sp. CRIB37 | EU683885 | 90 |
| 82_13 | KX451016 | Uncultured Chlamydiales bacterium clone GE10150water_C9 | JX083091 | 97 | domestic shower water | Neochlamydia sp. Trut23-12-2015_Venoge-Embouchure | LN995859 | 95 |
| 111_13 | KX451026 | Uncultured Chlamydiales bacterium clone HE210023_C12 | HQ721223 | 98 | nasopharyngeal samples from hospitalized children | Neochlamydia sp. Trut23-12-2015_Venoge-Embouchure | LN995859 | 98 |
| 96_13 | KX451020 | Uncultured Chlamydiales bacterium clone VS30044 | HQ721235 | 98 | nasopharyngeal samples from hospitalized children | Neochlamydia sp. Trut23-12-2015_Venoge-Embouchure | LN995859 | 96 |
| 90_13 | KX451018 | Neochlamydia sp. Trut23-12-2015_Venoge-Embouchure | LN995859 | 96 | Salmo trutta gills | Neochlamydia sp. Trut23-12-2015_Venoge-Embouchure | LN995859 | 96 |
| 144_13 | KX451036 | Uncultured Chlamydiales bacterium clone GE11093water | JX083098 | 96 | domestic shower water | Parachlamydia acanthamoebae strain Leol | AB812094 | 95 |
| 23_14 | KX451008 | Parachlamydia acanthamoebae | AB812094 | 97 | hot spring amoeba | Parachlamydia acanthamoebae strain Leol | AB812094 | 97 |
| 34_14 | KX451009 | Parachlamydia acanthamoebae | AB812094 | 96 | hot spring amoeba | Parachlamydia acanthamoebae strain Leol | AB812094 | 96 |
| 79_13 | KX451014 | Uncultured Chlamydiales bacterium clone GE11061 | HQ721207 | 94 | nasopharyngeal samples from hospitalized children | Parachlamydia acanthamoebae strain Leol | AB812094 | 91 |
| 86_13 | KX451017 | Uncultured Chlamydiales bacterium clone HE210023_C12 | HQ721223 | 99 | nasopharyngeal samples from hospitalized children | Parachlamydia acanthamoebae strain Leol | AB812094 | 97 |
| 134_13 | KX451034 | Uncultured Chlamydiales bacterium clone HE210032 | HQ721224 | 99 | nasopharyngeal samples from hospitalized children | Parachlamydia acanthamoebae strain Leol | AB812094 | 96 |
| 113_13 | KX451027 | Uncultured Chlamydiales bacterium clone 39IR | KF651170 | 97 | bovine rectal swab | Parachlamydia acanthamoebae strain Leol | AB812094 | 96 |
| 114_13 | KX451028 | Uncultured Chlamydiales bacterium clone 18IR | KF651171 | 94 | bovine rectal swab | Parachlamydia acanthamoebae strain Leol | AB812094 | 92 |
| 91_13 | KX451019 | Uncultured Chlamydiales bacterium clone GE10047 | HQ721195 | 94 | nasopharyngeal samples from hospitalized children | Parachlamydia acanthamoebae strain NS2 | JN051144 | 92 |
| 110_13 | KX451025 | Uncultured Chlamydiales bacterium clone GE10170water | JX083094 | 97 | domestic shower water | Parachlamydiaceae bacterium CRIB38 | EU683886 | 95 |
| 9_13 | KX451007 | Uncultured Chlamydiales bacterium clone 39IR | KF651170 | 94 | raw surface water | Parachlamydiaceae bacterium CRIB38 | EU683886 | 93% |
| T3_14 | KX451039 | Uncultured Chlamydiales bacterium clone 20IR | KF651172 | 95 | bovine rectal swab | Parachlamydiaceae bacterium CRIB38 | EU683886 | 93 |
| 105_13 | KX451023 | Parachlamydiaceae bacterium CRIB38 | EU683886 | 95 | raw surface water | Parachlamydiaceae bacterium CRIB38 | EU683886 | 95 |
| 58_14 | KX451012 | Uncultured Chlamydiales bacterium clone HE210050 | HQ721227 | 98 | nasopharyngeal samples from hospitalized children | Candidatus Syngnamydia salmonis isolate Ho-2008 | KF768763 | 90 |
| 102_13 | KX451021 | Uncultured Chlamydiales bacterium clone GE10047 | HQ721195 | 95 | nasopharyngeal samples from hospitalized children | Criblamydia sequanensis strain CRIB-18 | NR_115696 | 94 |
| T2_14 | KX451038 | Uncultured Chlamydiales bacterium clone GE10047 | HQ721195 | 96 | nasopharyngeal samples from hospitalized children | Criblamydia sequanensis strain CRIB-18 | NR_115696 | 93 |
| 5_14 | KX451006 | Uncultured Chlamydiales bacterium clone HE20074 | HQ721240 | 98 | nasopharyngeal samples from hospitalized children" | Criblamydia sequanensis strain CRIB-18 | NR_115696 | 94 |
| 122_13 | KX451031 | Uncultured Chlamydiales bacterium clone GE10064biof | JX083081 | 97 | domestic shower biofilm | Estrella lausannensis strain CRIB 30 | EU074225 | 89 |
| 117_13 | KX451029 | Uncultured Chlamydiales bacterium clone 39IR | KF651170 | 92 | bovine rectal swab | Waddlia chondrophila WSU 86-1044 | NR_074886 | 91 |
| 131_13 | KX451032 | Candidatus Rhabdochlamydia CLONE CLON20100330_02-17 | KF720713 | 97 | Oedothorax gibbosus | Candidatus Rhabdochlamydia porcellionis strain 15C | HF933203 | 96 |
| 62_13 | KX451013 | Uncultured Chlamydiales bacterium clone P2D4 | JQ860079 | 99 | Ixodes ricinus | Candidatus Rhabdochlamydia porcellionis strain 15C | HF933203 | 98% |
| 149_13 | KX451037 | Uncultured Chlamydiales bacterium clone P2D4 | JQ860079 | 99 | Ixodes ricinus | Candidatus Rhabdochlamydia porcellionis strain 15C | HF933203 | 98 |
| **Suspected tick bite** | | | | | | | | |
| **Skin Biopsy Number** | **GenBank Accession No.** | **Best BLAST Hit** | **Accession** | **Identity %** | **Host** | **Most Identical Established *Chlamydiales* Strain** | **Accession** | **Identity %** |
| 12-87 | KX451076 | Uncultured Chlamydiales bacterium clone P2D4 | JQ860079 | 98 | Ixodes ricinus | Candidatus Rhabdochlamydia porcellionis strain 15C | HF933203 | 97 |
| 14-23 | KX451107 | Uncultured Chlamydiales bacterium clone GE10064biof | JX083081 | 91 | domestic shower biofilm | Candidatus Rhabdochlamydia porcellionis strain 15C | HF933203 | 87 |
| 13-27 | KX451094 | Rhabdochlamydiaceae bacterium cvE55 | FJ976100 | 87 | fresh water | Rhabdochlamydiaceae bacterium cvE55 | FJ976100 | 87 |
| 14-55 | KX451109 | Uncultured Candidatus Rhabdochlamydia sp. clone CN808 | EU090709 | 89 | clinical respiratory samples of | Rhabdochlamydiaceae bacterium NS3 | JN051145 | 88 |
| 13-33 | KX451095 | Uncultured Chlamydiales bacterium clone GE10088water | JX083084 | 91 | domestic shower water | Rhabdochlamydiaceae bacterium NS3 | JN051145 | 90 |
| 13-07 | KX451087 | Uncultured Chlamydiales bacterium clone GE10064biof | JX083081 | 96 | domestic shower biofilm | Candidatus Renichlamydia lutjani clone ELO | JN167597 | 88 |
| 12-114 | KX451083 | Chlamydiales bacterium NS16 | JN606076 | 88 | human nasal sample | Rhabdochlamydiaceae bacterium cvE99 | JF513057 | 87 |
| 12-88 | KX451077 | Uncultured Candidatus Rhabdochlamydia sp. clone CN808 | EU090709 | 89 | clinical respiratory samples | Candidatus Fritschea bemisiae strain Falk |  | 86 |
| 13-03 | KX451086 | Uncultured Chlamydiales bacterium clone GE10016biof | JX083073 | 93 | domestic shower biofilm | Simkania negevensis strain Z | NR_074932 | 88 |
| 13-120 | KX451102 | Uncultured Chlamydiales bacterium clone P2H10-2 | JQ860083 | 98 | Ixodes ricinus | Simkania negevensis strain Z | NR_074932 | 90 |
| 12-96 | KX451116 | Uncultured Chlamydiales bacterium clone GE10016biof | JX083073 | 91 | domestic shower biofilm | Simkania negevensis strain Z | NR_074932 | 89 |
| 12-107 | KX451119 | Uncultured Chlamydiales bacterium clone 18IR | KF651171 | 89 | bovine rectal swab | Neochlamydia sp. Trut23-12-2015_Venoge-Embouchure | LN995859 | 87 |
| 12-81 | KX451110 | Uncultured Chlamydiales bacterium clone VS30055biof | JX083116 | 89 | domestic shower biofilm | Parachlamydia acanthamoebae strain Leol | AB812094 | 89 |
| 12-83 | KX451075 | Uncultured Chlamydiales bacterium clone GE10088water | JX083084 | 97 | domestic shower water | Parachlamydiaceae bacterium HS-T3 | AB812093 | 88 |
| 12-92 | KX451114 | Uncultured Chlamydiales bacterium clone GE10150water_C9 | JX083091 | 91 | domestic shower water | Candidatus Protochlamydia sp. cvE14 | FJ976093 | 91 |
| 13-13 | KX451091 | Chlamydiales bacterium CRIB 32 | EU363464 | 93 | biofilm (water network) | Candidatus Protochlamydia sp. cvE14 | FJ976093 | 87 |
| 12-120 | KX451084 | Uncultured Chlamydiales bacterium clone HE20036biof | JX083104 | 95 | domestic shower biofilm | Candidatus Protochlamydia sp. cvE14 | FJ976093 | 88 |
| 12-97 | KX451080 | Uncultured Chlamydiae bacterium clone Upland_40_6069 | JF986771 | 97 | upland cropland soils | Candidatus Protochlamydia sp. cvE14 | FJ976093 | 90 |
| 13-25b | KX451092 | Uncultured Chlamydiales bacterium clone GE10016water | JX083072 | 98 | domestic shower water | Candidatus Metachlamydia lacustris strain CHSL | GQ221847 | 93 |
| 13-107 | KX451099 | Uncultured Chlamydiae bacterium clone Upland_500_9569 | JF990268 | 97 | upland cropland soils | Neochlamydia hartmannellae strain A1Hsp | NR_025037 | 91 |
| 12-104 | KX451081 | Uncultured Chlamydiales bacterium clone GE10016biof | JX083073 | 93 | domestic shower biofilm | Estrella lausannensis strain CRIB 30 | EU074225 | 88 |
| 14-02 | KX451104 | Chlamydiales bacterium CRIB 32 | EU363464 | 96 | biofilm (water network) | Estrella lausannensis strain CRIB 30 | EU074225 | 89 |
| 13-04 | KX451113 | Uncultured Chlamydiales bacterium clone HE20032water | JX083102 | 84 | domestic shower biofilm | Estrella lausannensis strain CRIB 30 | EU074225 | 87 |
| 13-101 | KX451097 | Chlamydiales bacterium CRIB33 | EU683887 | 91 | raw surface water | Estrella lausannensis strain CRIB 30 | EU074225 | 88 |
| 14-41 | KX451108 | Uncultured Chlamydiales bacterium clone GE10016water | JX083072 | 97 | domestic shower water | Estrella lausannensis strain CRIB 30 | EU074225 | 89 |
| 12-103 | KX451120 | Uncultured Chlamydiales bacterium clone GE10016water | LN831098 | 93 | domestic shower water | Estrella lausannensis strain CRIB 30 | EU074225 | 88 |
| 13-23 | KX451118 | Uncultured Chlamydiales bacterium clone GE10068biof | JX083083 | 90 | domestic shower biofilm | Candidatus Rhabdochlamydia crassificans clone P1s-222 | GQ287585 | 89 |
| 12-94 | KX451079 | Uncultured Chlamydiales bacterium clone P2D4 | JQ860079 | 96 | Ixodes ricinus | Candidatus Rhabdochlamydia sp. cvE88 | JF513056 | 95 |
| 13-125 | KX451103 | Chlamydiales bacterium NS16 | JN606076 | 96 | human nasal sample | Rhabdochlamydiaceae bacterium cvE99 | JF513057 | 87 |
| 14-09 | KX451105 | Uncultured Chlamydia sp. clone PRPR85 | DQ903997 | 92 | Hawaiian marine sponge | Simkania negevensis strain Z | NR_074932 | 90 |
| 13-112 | KX451100 | Uncultured Chlamydiales bacterium clone GE10016biof | JX083073 | 94 | domestic shower biofilm | Simkania negevensis strain Z | NR_074932 | 84 |
| 13-02 | KX451111 | Uncultured Chlamydiales bacterium clone GE10027water_F2 | JX083075 | 93 | domestic shower water | Simkania negevensis strain Z | NR_074932 | 88 |
| 13-25 | KX451093 | Uncultured Chlamydiales bacterium clone P-5 | AF364565 | 95 | waste water treatment plants | Estrella lausannensis strain CRIB 30 | EU074225 | 90 |
| 13-102 | KX451098 | Estrella lausannensis strain CRIB 30 | EU074225 | 87 | water treatment plant | Estrella lausannensis strain CRIB 30 | EU074225 | 87 |
| 13-08 | KX451088 | Uncultured Chlamydiales bacterium clone GE10148water | JX083089 | 96 | domestic shower biofilm | Estrella lausannensis strain CRIB 30 | EU074225 | 88 |
| 10-101 | KX451073 | Uncultured Chlamydiales bacterium clone HE210011biof | JX083108 | 95 | domestic shower biofilm | Estrella lausannensis strain CRIB 30 | EU074225 | 92 |
| 12-102 | KX451121 | Uncultured Chlamydiales bacterium clone GE10016biof | JX083073 | 85 | domestic shower biofilm | Estrella lausannensis strain CRIB 30 | EU074225 | 88 |
| 13-05 | KX451117 | Uncultured Chlamydia sp. clone PRPR85 | DQ903997 | 90 | Hawaiian marine sponge | Estrella lausannensis strain CRIB 30 | EU074225 | 89 |
| 13-99 | KX451096 | Uncultured Chlamydiales bacterium clone HE210012biof | JX083110 | 96 | domestic shower biofilm | Estrella lausannensis strain CRIB 30 | EU074225 | 88 |
| 13-12 | KX451112 | Uncultured Chlamydiales bacterium clone HE20036biof | JX083104 | 87 | domestic shower biofilm | Estrella sp. CRIB 31 | EU363463 | 86 |
| 13-28 | KX451115 | Uncultured Chlamydiales bacterium clone GE10027water_F2 | JX083075 | 87 | domestic shower water | Estrella lausannensis strain CRIB 30 | EU074225 | 87 |
| 13-117 | KX451101 | Uncultured Chlamydiales bacterium clone GE10027water_F2 | JX083075 | 96 | domestic shower water | Estrella lausannensis strain CRIB 30 | EU074225 | 90 |
| 12-108 | KX451082 | Uncultured Chlamydiales bacterium clone GE10016biof | JX083073 | 93 | domestic shower biofilm | Estrella lausannensis strain CRIB 30 | EU074225 | 89 |
| 12-91 | KX451078 | Uncultured Chlamydiales bacterium clone AAL4c65 | KC902452 | 92 | Ace Lake / antarctica | Candidatus Protochlamydia sp. cvE14 | FJ976093 | 87 |
| 14-13 | KX451106 | Uncultured Chlamydiales bacterium clone HE210023biof | JX083111 | 96 | domestic shower biofilm | Neochlamydia hartmannellae strain A1Hsp | NR_025037 | 91 |
| 13-09 | KX451089 | Uncultured Chlamydiales bacterium clone HE210012biof | JX083110 | 94 | domestic shower biofilm | Candidatus Protochlamydia sp. cvE14 | FJ976093 | 88 |
| 13-01 | KX451085 | Uncultured Chlamydiales bacterium clone HE210012biof | JX083110 | 96 | domestic shower biofilm | Candidatus Protochlamydia sp. cvE14 | FJ976093 | 89 |
| 13-10 | KX451090 | Uncultured Chlamydiales bacterium clone P2H10-2 | JQ860083 | 96 | Ixodes ricinus | Neochlamydia hartmannellae strain A1Hsp | NR_025037 | 91 |
| 12-82 | KX451074 | Uncultured Chlamydiales bacterium clone VS30055biof | JX083116 | 93 | domestic shower biofilm | Parachlamydia acanthamoebae strain NS2 | JN051144 | 91 |
| **Healthy Individuals** | | | | | | | | |
| **Skin Biopsy Identification** | **GenBank Accession No.** | **Best BLAST Hit** | **Accession** | **Identity %** | **Host** | **Most Identical Established *Chlamydiales* Strain** | **Accession** | **Identity %** |
| KaLa | KX451061 | Simkania negevensis strain Z | NR_074932 | 89 |  | Simkania negevensis strain Z | NR_074932 | 89 |
| AhEe | KX451056 | Uncultured Chlamydiales bacterium clone P-5 | AF364565 | 95 | waste water treatment plants | Parachlamydiaceae bacterium HS-T3 | AB812093 | 93 |
| HoKa | KX451058 | Parachlamydiaceae bacterium CRIB38 | EU683886 | 93 | raw surface water | Parachlamydiaceae bacterium CRIB38 | EU683886 | 93 |
| MaRi | KX451064 | Parachlamydiaceae bacterium CRIB38 | EU683886 | 95 | raw surface water | Parachlamydiaceae bacterium CRIB38 | EU683886 | 95 |
| RiAl | KX451066 | Parachlamydiaceae bacterium CRIB38 | EU683886 | 95 | raw surface water | Parachlamydiaceae bacterium CRIB38 | EU683886 | 95 |
| RoMi | KX451067 | Uncultured Chlamydiales bacterium clone HE210023_C12 | HQ721223 | 94 | nasopharyngeal samples | Parachlamydiaceae bacterium CRIB38 | EU683886 | 94 |
| JoAk | KX451060 | Uncultured Chlamydiales bacterium clone 21IR | KF651173 | 90 | bovine rectal swab | Parachlamydia acanthamoebae strain Leol | AB812094 | 88 |
| RaJu | KX451065 | Uncultured Neochlamydia sp. clone Depth_24to36-11 | JQ288593 | 98 | drinking water treatment plant | Neochlamydia hartmannellae strain A1Hsp | NR_025037 | 92 |
| AuMi | KX451057 | Uncultured Chlamydiales bacterium clone P-5 | AF364565 | 93 | waste water treatment plants | Candidatus Mesochlamydia elodeae strain KV | JN112799 | 87 |
| LaKi | KX451063 | Uncultured Chlamydiales bacterium clone GE10027water_F2 | JX083075 | 95 | domestic shower water | Candidatus Mesochlamydia elodeae strain KV | JN112799 | 87 |
| AgYl | KX451071 | Uncultured Chlamydiales bacterium clone HE210045_C6 | HQ721226 | 91 | nasopharyngeal samples | Neochlamydia sp. Trut23-12-2015_Venoge-Embouchure | LN995859 | 90 |
| SaRi | KX451069 | Uncultured Chlamydiales bacterium clone P-5 | AF364565 | 94 | waste water treatment plants | Candidatus Metachlamydia lacustris strain CHSL | GQ221847 | 91 |
| LeMo | KX451070 | Uncultured Chlamydiales bacterium clone HE20032water | JX083102 | 90 | domestic shower water | Estrella lausannensis strain CRIB 30 | EU074225 | 89 |
| KuRa | KX451062 | Uncultured Chlamydiales bacterium clone HE210012biof | JX083110 | 93 | domestic shower biofilm | Estrella lausannensis strain CRIB 30 | EU074225 | 89 |
| HuHe | KX451059 | Uncultured Chlamydiales bacterium clone GE10027water_F2 | JX083075 | 95 | domestic shower water | Estrella lausannensis strain CRIB 30 | EU074225 | 89 |
| PuVer | KX451072 | Uncultured Chlamydiales bacterium clone GE11103 | HQ721213 | 97 | nasopharyngeal samples | Estrella lausannensis strain CRIB 30 | EU074225 | 96 |
| RuVe | KX451068 | Uncultured Chlamydiales bacterium clone GE10047 | HQ721195 | 95 | nasopharyngeal samples | Criblamydia sequanensis strain CRIB-18 | NR_115696 | 91 |

**Figure S1.** Maximum likelihood tree with 100 bootstrap replicates of 16S sequences analyzed in this study. Bootstrap support values ≥ 50 are shown in the ML tree. Raw sequences were trimmed for primers and poor quality regions and subsequently aligned with sequences downloaded from GenBank. Software Geneious (Geneious version 6.1 created by Biomatters. Available from http://www.geneious.com) was used to draw a maximum likelihood tree using PHYML plugin (Guindon & Gascuel 2003; Kearse et al. 2012; PHYML plugin was developed by V. Lefort, J. Heled, S. Guindon and the Geneious team). Sequences downloaded from GenBank in the tree are indicated with taxonomic identification and accession number.
